# Comparative Effectiveness of Autologous Blood Clot Therapy (ActiGraft), Autologous Micrograft Therapy (Rigenera), and Advanced Wound Dressings for Refractory Chronic Lower Limb Ulcers: A Real-World Evidence Study

**DOI:** 10.3390/jcm15051902

**Published:** 2026-03-02

**Authors:** Muhammad Khatib, Dror Robinson, Eitan Lavon, Feras Qawasmi, Waseem Abu Rashed, Hamza Murad, Yaffa Maximov, Assil Mahamid, Mustafa Yassin

**Affiliations:** 1 Department of Orthopedics, Hasharon Hospital, Rabin Medical Center, Petah Tikva 4941492, Israelmustafay@clalit.org.il (M.Y.); 2 Gray Faculty of Medicine, Tel Aviv University, Tel Aviv 6997801, Israel; 3 Management Department, Clalit Health Services, Rabin Medical Center, Petah Tikva 4941492, Israel

**Keywords:** chronic wounds, autologous blood clot therapy, autologous micrograft therapy, ActiGraft, Rigenera, diabetic foot ulcers, wound healing, real-world evidence, platelet-rich plasma, number needed to treat

## Abstract

**Background/Objectives**: Chronic lower limb ulcers represent a significant clinical challenge, with conventional therapies achieving healing in only 30–40% of complex cases. This study evaluated the comparative effectiveness of autologous blood clot therapy (ActiGraft, delivering platelet- and leukocyte-derived growth factors) and autologous micrograft therapy (Rigenera, containing viable progenitor cells) versus advanced wound dressings for refractory chronic wounds. **Methods**: This retrospective analysis of a prospectively collected, non-randomized clinical cohort included 132 patients with chronic lower limb ulcers refractory to prior therapy, who were treated between 2019 and 2024 at a single wound care center. The patients received ActiGraft (n = 32), Rigenera (n = 33), or advanced wound dressings (n = 67) based on their choice after informed discussion. The primary outcome was complete wound closure at 52 weeks. Multivariable Poisson regression with robust variance was performed, adjusting for baseline wound area (log-transformed), chronic renal failure, age, and peripheral vascular disease. Cox proportional hazards was used to model time to closure. Bonferroni correction (threshold *p* < 0.0167) was applied for three pairwise comparisons. This study was not pre-registered, and the results should be considered hypothesis-generating. **Results**: Unadjusted wound closure rates were 68.8% (ActiGraft; RR = 1.71, 95% CI: 1.17–2.48, *p* = 0.015), 60.6% (Rigenera; RR = 1.50, 95% CI: 1.01–2.25, *p* = 0.089), and 40.3% (advanced dressings). After multivariable adjustment, ActiGraft showed attenuated benefit (adjusted RR = 1.38, 95% CI: 0.86–2.21, *p* = 0.179), while the beneficial effect of Rigenera became non-significant (adjusted RR = 1.19, 95% CI: 0.73–1.94, *p* = 0.488). However, the adjusted Cox regression revealed significantly faster healing for ActiGraft (HR = 10.67, 95% CI: 4.17–27.30, *p* < 0.001) and Rigenera (HR = 4.12, 95% CI: 1.75–9.73, *p* = 0.001). Sensitivity analyses restricted to comparable wound sizes (≤10 cm^2^) showed a consistent direction of effect (ActiGraft 71.4% vs. Advanced 37.5%). Infection rates were lower in the autologous therapy groups (0–3.0% vs. 11.9%; Fisher’s exact *p* = 0.006). **Conclusions**: ActiGraft autologous blood clot therapy showed trends toward superior wound closure and demonstrated significantly faster healing compared to advanced dressings in patients with refractory chronic lower limb ulcers, with autologous micrograft therapy (Rigenera) showing intermediate results. Significant baseline imbalances in wound size limit causal inference from the closure rate comparisons. These hypothesis-generating findings from a non-randomized cohort warrant confirmation in adequately powered randomized controlled trials with stratification by wound characteristics.

## 1. Introduction

Chronic wounds, particularly diabetic foot ulcers (DFUs), represent a significant global healthcare burden affecting approximately 15–25% of diabetic patients during their lifetime [1,2]. With an estimated 537 million adults living with diabetes in 2021, projected to reach 783 million by 2045, the prevalence of DFUs continues to rise worldwide, with annual treatment costs exceeding $25–50 billion globally [3,4]. Despite substantial advances in wound care technology over the past two decades, conventional therapies—including advanced dressings, negative pressure wound therapy, and bioengineered skin substitutes—achieve complete healing in only 30–40% of complex or refractory cases [5,6]. This therapeutic gap results in substantial morbidity: Chronic wounds are the leading cause of non-traumatic lower extremity amputation, significantly reduce quality of life, and are associated with five-year mortality rates of 40–50%, figures that are comparable to many malignancies such as colon or breast cancer [7,8].

The pathophysiology of chronic wounds involves a complex interplay of impaired angiogenesis, persistent inflammation, reduced growth factor activity, bacterial biofilm formation, and compromised cellular migration and proliferation [9,10]. In diabetic patients, these deficits are compounded by peripheral neuropathy, peripheral arterial disease, and hyperglycemia-induced cellular dysfunction, creating a self-perpetuating cycle of impaired healing [11]. Traditional wound care approaches, while addressing some of these factors, often fail to restore the coordinated biological processes necessary for tissue regeneration in severely compromised wounds [12]. Recent research highlights the need for therapies that target multiple pathophysiological mechanisms simultaneously, moving beyond passive wound coverage toward active biological stimulation [13,14].

Autologous therapies have emerged as promising alternatives for refractory wounds by leveraging the patient’s own biological resources to stimulate tissue regeneration [15,16]. Two such approaches with distinct mechanisms merit comparative evaluation. ActiGraft (RedDress Ltd., Pardes Hanna, Israel) utilizes autologous whole blood activated by kaolin (a contact activator of the coagulation cascade) to create a bioactive clot scaffold. This product delivers platelet- and leukocyte-derived growth factors (including PDGF, TGF-β, VEGF, and EGF) within a natural fibrin matrix, promoting angiogenesis, cellular migration, and tissue remodeling [17,18]. Importantly, ActiGraft is classified as an autologous blood clot therapy rather than a cell-based regenerative therapy: It utilizes non-enriched whole blood (platelet concentration ~200,000/µL, equivalent to circulating levels), functioning as a biologic dressing comparable to platelet-rich plasma (PRP) products but without centrifugation or concentration enrichment [19,20]. Rigenera (Human Brain Wave, Turin, Italy) employs a fundamentally different approach through mechanical disaggregation of autologous connective tissue to generate micrografts containing viable progenitor cells with multipotent differentiation capacity, including mesenchymal stem cells, fibroblasts, and extracellular matrix components [21,22]. This cell-based therapy provides direct cellular replenishment to the wound bed.

The mechanistic distinction between these approaches has clinical implications. ActiGraft’s repeated application protocol (4–8 weekly sessions) provides sustained growth factor delivery, potentially maintaining a prolonged anabolic wound environment; however, its efficacy relies on the patient’s existing growth factor production capacity, which may be impaired in diabetes [17]. Rigenera’s single-application protocol delivers viable progenitor cells capable of autonomous proliferation and differentiation, potentially addressing cellular deficiency directly; however, the single-dose approach may limit sustained biological stimulation [21]. These mechanistic differences suggest potentially differential effectiveness in different wound phenotypes (e.g., ischemic versus neuropathic ulcers), although comparative data remain limited.

While individual studies suggest efficacy for both ActiGraft and Rigenera [23,24], direct comparative data between these autologous approaches and standard care in real-world clinical settings remain sparse. Real-world evidence (RWE) studies provide valuable insights into treatment effectiveness under routine clinical conditions and complement randomized controlled trials by capturing outcomes in diverse patient populations [25,26]. This retrospective cohort study compared wound closure rates, time to healing, and complications between ActiGraft, Rigenera, and advanced wound dressings in patients with chronic lower limb ulcers refractory to prior conventional therapy. Pre-specified adjusted analyses accounted for baseline imbalances to strengthen causal inference within the limitations of a non-randomized design.

## 2. Materials and Methods

### 2.1. Study Design and Setting

This retrospective analysis of a non-randomized, prospectively collected clinical cohort was conducted at the specialized wound care clinic of Hasharon Hospital, Rabin Medical Center, Israel. Patients received treatment as part of routine clinical care between January 2019 and December 2024. The hybrid design involved prospective data collection standardized through electronic health records (Clalit Health Services, Tel Aviv, Israel) to minimize recall bias, with retrospective de-identified analysis. Treatment allocation followed a patient-choice design reflecting real-world clinical practice, where patients selected therapy after a comprehensive informed discussion of available options, associated procedures, expected outcomes, and potential risks. This design introduced potential confounding by indication, as well as motivation and socioeconomic confounding, which was addressed through multivariable adjustment and sensitivity analyses (Section 2.7). This study was not pre-registered on a clinical trial registry, and all analyses should be considered hypothesis-generating.

Retrospective analyses of de-identified clinical data were approved by the Institutional Review Board of Rabin Medical Center (Protocol RMC-0634-25, approved 3 July 2025). This study was conducted in accordance with the Declaration of Helsinki. Written informed consent for treatment was obtained from all patients; the IRB granted a waiver of additional consent for retrospective chart review given the minimal risk and de-identified nature of the analysis. This study followed the STROBE (Strengthening the Reporting of Observational Studies in Epidemiology) checklist for the reporting of observational studies (Supplementary Materials).

### 2.2. Participants

The inclusion criteria were as follows: (1) chronic lower limb ulcer (duration > 4 weeks) without evidence of healing progression; (2) documented failure of prior conventional therapy including at least one advanced wound dressing; (3) adequate arterial perfusion (ankle–brachial index [ABI] > 0.4 or toe pressure > 40 mmHg); and (4) age ≥ 18 years. The exclusion criteria were as follows: (1) active wound infection requiring systemic antibiotics at enrollment; (2) osteomyelitis confirmed by imaging or biopsy; (3) malignancy at the wound site; (4) immunosuppressive therapy (>10 mg/day prednisone equivalent); (5) uncontrolled diabetes (HbA1c > 12%); and (6) severe malnutrition (albumin < 2.5 g/dL). Of the 156 patients screened, 24 were excluded: 10 for active infection or osteomyelitis, 8 for peripheral arterial disease (ABI ≤ 0.4), and 6 for refusal to participate. Exclusions were balanced across treatment groups.

### 2.3. Classification of Interventions

Interventions were classified based on their biological mechanism of action. ActiGraft^Pro@^ (RedDress, 822 A1A North Ste 310, Ponte Vedra Beach, FL 32082, USA) is classified as an autologous blood clot therapy utilizing platelet- and leukocyte-derived growth factors delivered in a fibrin matrix. It uses non-enriched whole blood (platelet concentration ~200,000/µL, equivalent to circulating levels) without centrifugation and is mechanistically comparable to PRP products [19,20]. Rigenera^@^ MicroGrafting Technology (Human Brain Wave (HBW) Srl, C.so Galileo Ferraris, 63 10128 Turin, Italy) is classified as an autologous micrograft (cell-based) therapy containing viable progenitor cells, including mesenchymal stem cells, fibroblasts, and extracellular matrix components [21]. Advanced wound dressings are passive moisture-management dressings providing an optimal wound environment without direct biological activity. The term “autologous therapies” is used throughout to collectively describe ActiGraft and Rigenera, acknowledging their distinct mechanisms.

### 2.4. Interventions

ActiGraft (Autologous Blood Clot Therapy): ActiGraft comprised weekly applications (4–8 sessions, median = 5; distribution: 4 sessions: n = 9; 5 sessions: n = 11; 6 sessions: n = 4; 7 sessions: n = 6; 8 sessions: n = 2) of a bioactive clot created from 20 mL of autologous venous blood activated with kaolin powder. The procedure involved venipuncture, blood collection into the proprietary device, incubation for 5–10 min to allow fibrin clot formation, wound bed preparation with sharp debridement, and clot application with non-adherent secondary dressing. No biological characterization of the clot (growth factor concentrations, platelet activation markers, or leukocyte counts) was performed, which is acknowledged as a limitation. The kaolin dose and formulation are proprietary to RedDress Ltd.

Rigenera (Autologous Micrograft Therapy): Rigenera comprised a single application of autologous skin micrografts. The procedure involved local anesthesia, collection of 2–4 punch biopsies (2 mm diameter) from the perilesional or posterior auricular area, mechanical processing using the Rigeneracons device to generate micrografts, wound bed preparation with sharp debridement, and uniform application of micrograft suspension. Cell viability and progenitor cell quantification (e.g., CD34+, CD90+, CD73+, and CD105+ flow cytometry) were not performed in this clinical setting, which is acknowledged as a limitation.

Advanced Wound Dressings (Control): Standard wound care with dressings was selected based on wound characteristics: hydrocolloids, polyurethane foams, calcium alginates, hydrogels, or antimicrobial dressings (silver-containing) as clinically indicated. Dressing changes occurred 2–3 times weekly.

Standardized Co-interventions: All groups received sharp debridement as indicated, pressure offloading for DFUs (removable cast walkers or therapeutic footwear per IWGDF guidelines), compression therapy (30–40 mmHg graduated stockings) for venous ulcers, glycemic optimization targeting HbA1c < 8%, and nutritional counseling. The unequal treatment frequency (multiple ActiGraft sessions vs. a single Rigenera application) introduces potential performance bias, as discussed in the limitations.

### 2.5. Wound Assessment and Classification

Wounds were assessed at baseline and at 6, 12, 26, and 52 weeks using standardized digital photography with a calibrated ruler for scale. The wound area was measured via digital planimetry using ImageJ software version 1.54p (NIH, Bethesda, MD). Wound depth was measured in millimeters at the deepest point using a sterile probe. Wound volume was approximated as area × depth/10 (cm^3^); this simplified formula assumes a conical wound geometry and is acknowledged as an estimate that may oversimplify irregular wound shapes [27]. Wounds that achieved complete closure before 52 weeks continued to be assessed at scheduled visits; healed wounds were carried forward as zero area in longitudinal analyses.

Infection was defined by clinical signs (erythema extending > 2 cm from wound margin, purulent discharge, warmth, and pain/tenderness) or positive probe-to-bone test, requiring initiation of systemic antibiotic therapy. Neuropathy was assessed using 10 g Semmes–Weinstein monofilament testing at four plantar sites. Wounds were classified using the SINBAD (Site, Ischemia, Neuropathy, Bacterial infection, Area, Depth) scoring system [28], with each component scored 0 or 1 (total range of 0–6). Peripheral arterial status was categorized by ABI as normal (≥1.0), mild PAD (0.5–0.99), or severe PAD (<0.5). Outcome assessors (independent evaluators) were not blinded to treatment allocation; this was not feasible due to visible differences in treatment application. All wound photographs were reviewed by two independent clinicians, and discrepancies were resolved by consensus.

### 2.6. Outcomes

Primary Outcome: Complete wound closure at 52 weeks, defined as 100% epithelialization without drainage, confirmed at two consecutive visits 2–4 weeks apart by independent evaluators using standardized photographs.

Secondary Outcomes: (1) Time to complete wound closure, analyzed as a time-to-event outcome with non-healed wounds censored at last follow-up or 52 weeks; (2) percentage wound area reduction at 12, 26, and 52 weeks, reported without adjustment for baseline area in descriptive analyses; (3) complications, including wound infection requiring systemic antibiotics, hospitalization for wound-related causes, and major amputation (defined as above-ankle level, including transtibial and transfemoral).

### 2.7. Statistical Analysis

Sample size was calculated assuming 35% vs. 60% closure rates (α = 0.05, power = 80%, two-sided), requiring approximately 62 patients per arm. This study achieved adequate power (>80%) for the primary ActiGraft vs. advanced dressings comparison but was underpowered for the Rigenera subgroup comparison (estimated power ~60%), with a corresponding Type II error risk noted. Continuous variables are presented as mean ± SD or median (IQR); categorical variables are presented as frequencies (percentages). Baseline group comparisons used Kruskal–Wallis tests for continuous variables and chi-square or Fisher’s exact tests for categorical variables.

Primary Analysis: Unadjusted relative risk (RR) with 95% CI was calculated using the Katz log method. The number needed to treat (NNT) and absolute risk reduction (ARR) were computed. Three pairwise comparisons were performed (ActiGraft vs. advanced, Rigenera vs. advanced, and Combined autologous vs. advanced), with Bonferroni correction applied (adjusted significance threshold: *p* < 0.0167). The combined autologous analysis was pre-specified as exploratory, pooling two therapies with distinct mechanisms, and the results should be interpreted with this caveat.

Adjusted Analysis: Multivariable Poisson regression with log link and robust (Huber–White HC1) covariance was performed to estimate the adjusted RR for wound closure, including treatment group, log-transformed baseline wound area (log[area + 1] to address right skew), chronic renal failure (binary), age (continuous), and peripheral vascular disease (binary) as covariates. Cox proportional hazards regression was used to model time to wound closure with the same covariates, providing adjusted hazard ratios (HR) with 95% CI. The proportional hazards assumption was assessed using Schoenfeld residual plots.

Sensitivity Analyses: Analyses included (1) wound size-restricted analyses limited to patients with a baseline wound area of <15 cm^2^ and <10 cm^2^, where group overlap in wound size was greatest; (2) stratified analyses by wound size categories (<10, 10–20, >20 cm^2^) and by chronic renal failure status; (3) logistic regression for infection outcome with adjustment for wound size. These analyses were performed using R version 4.3.2 (R Foundation, Vienna, Austria) and Python 3.12 with statsmodels and lifelines libraries.

## 3. Results

### 3.1. Patient Enrollment and Baseline Characteristics

Of the 156 patients screened, 132 met the eligibility criteria and were enrolled: advanced dressings (n = 67), ActiGraft (n = 32), and Rigenera (n = 33). Follow-up completion was 98.5% (130/132; two patients were lost to follow-up, one each in the ActiGraft and Rigenera groups). The cohort had a mean age of 64.2 ± 11.8 years, 58.3% male, and 97.0% with diabetes. The groups were similar in age (*p* = 0.237), sex (*p* = 0.254), diabetes prevalence (*p* = 0.362), and SINBAD total scores (mean: 5.0–5.1; range: 5–6; *p* = 0.184).

However, clinically important baseline imbalances were present. Baseline wound areas differed significantly (*p* < 0.001): Advanced dressings had a median of 20.0 cm^2^ (IQR: 8.0–33.0) versus ActiGraft’s 9.0 cm^2^ (IQR: 6.5–9.5) and Rigenera’s 9.0 cm^2^ (IQR: 6.0–12.0). The wound area distribution shows minimal overlap between groups: No ActiGraft patients had wounds > 20 cm^2^, while 49.3% (33/67) of the advanced dressings patients did. Chronic renal failure was more prevalent in autologous therapy groups (ActiGraft: 43.8%; Rigenera: 45.5%) compared to the advanced dressings group (20.9%; *p* = 0.014). Peripheral vascular disease showed a non-significant trend (advanced: 38.8%; ActiGraft: 40.6%; Rigenera: 48.5%; *p* = 0.622). SINBAD scores showed limited variability: all patients had SINBAD = 5 (n = 121, 91.7%) or SINBAD = 6 (n = 11, 8.3%), limiting stratification by wound severity (Table 1).

### 3.2. Primary Outcome: Wound Closure at 52 Weeks

Complete wound closure rates at 52 weeks were as follows. Advanced dressings: 40.3% (27/67, 95% CI: 29.4–52.3%); ActiGraft: 68.8% (22/32, 95% CI: 51.4–82.0%); and Rigenera: 60.6% (20/33, 95% CI: 43.7–75.3%) (Table 2).

Unadjusted Analysis: ActiGraft demonstrated significantly higher wound closure compared to advanced dressings (RR = 1.71, 95% CI: 1.17–2.48, *p* = 0.015). The ARR was 28.5%, corresponding to NNT = 3.5. Rigenera showed a non-significant trend toward improvement (RR = 1.50, 95% CI: 1.01–2.25, *p* = 0.089; NNT = 4.9). Exploratory combined autologous therapy analysis showed 64.6% closure (42/65) versus 40.3% for advanced dressings (RR = 1.60, 95% CI: 1.13–2.27, *p* = 0.009; NNT = 4.1). After Bonferroni correction (threshold *p* < 0.0167), ActiGraft vs. advanced dressings (*p* = 0.015) remained significant, and the combined comparison (*p* = 0.009) remained significant, while Rigenera vs. advanced dressings (*p* = 0.089) was clearly non-significant.

Adjusted Analysis: After multivariable Poisson regression adjusting for log-transformed baseline wound area, chronic renal failure, age, and peripheral vascular disease, the treatment effects were attenuated: ActiGraft adjusted RR = 1.38 (95% CI: 0.86–2.21, *p* = 0.179); Rigenera adjusted RR = 1.19 (95% CI: 0.73–1.94, *p* = 0.488); and combined adjusted RR = 1.28 (95% CI: 0.83–1.98, *p* = 0.270). Chronic renal failure was an independent predictor of wound closure (adjusted RR = 1.60, 95% CI: 1.14–2.24, *p* = 0.007). The attenuation of treatment effects after adjustment is consistent with confounding by indication, wherein patients with smaller wounds preferentially selected autologous therapies.

### 3.3. Time to Wound Closure

**Figure 1:**
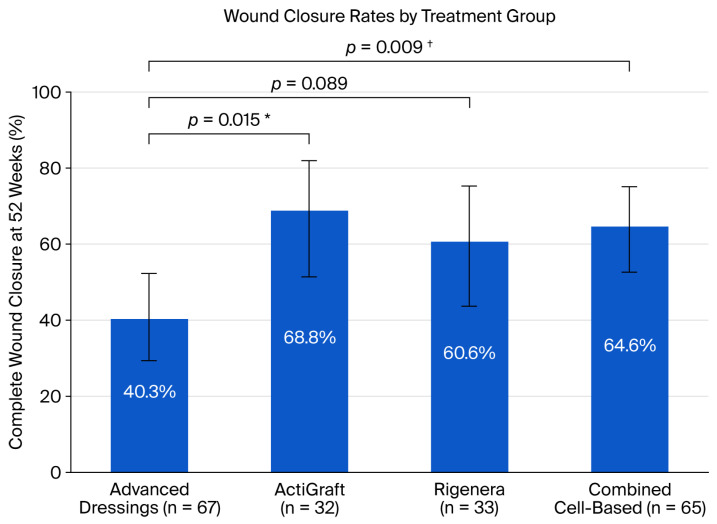
Kaplan–Meier curves for time to complete wound closure. The asterisk denotes significant results. The dagger denotes highly significant results.

**Figure 2:**
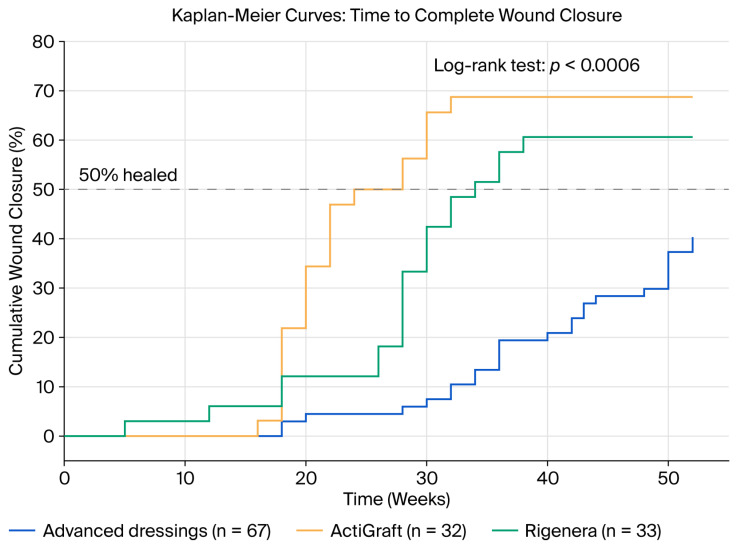
Kaplan-Meier survival curves comparing the three treatment modalities.

Kaplan–Meier analysis demonstrated significantly faster wound closure with autologous therapies (overall log-rank *p* = 0.0006; Figure 1). The median time to closure among healed patients was 21 weeks for ActiGraft (95% CI: 18–27), 28 weeks for Rigenera (95% CI: 26–32), and 40 weeks for advanced dressings (95% CI: 33–49) (Figure 2). Non-healed wounds were censored at 52 weeks or at the last follow-up for the two patients lost to follow-up.

**Figure 3:**
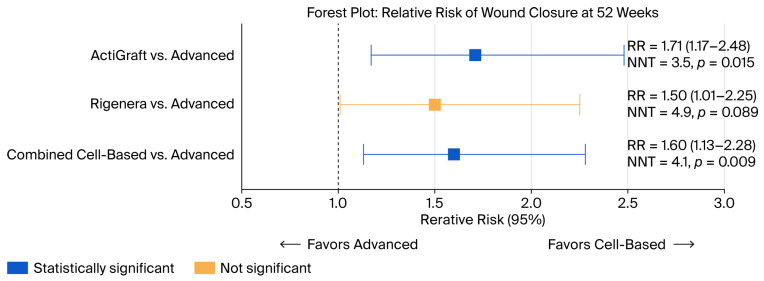
Forest Plot: Relative Risk of Wound Closure at 52 Weeks.

Adjusted Cox proportional hazards regression (Figure 3) demonstrated significantly faster healing for both autologous therapies after adjustment for baseline wound area, CRF, age, and PVD (ActiGraft: adjusted HR = 10.67 (95% CI: 4.17–27.30, *p* < 0.001); Rigenera: adjusted HR = 4.12 (95% CI: 1.75–9.73, *p* = 0.001)). These hazard ratios indicate substantially accelerated healing trajectories for autologous therapies. Neither baseline wound area (HR = 0.74, 95% CI: 0.40–1.38, *p* = 0.348) nor CRF (HR = 1.45, 95% CI: 0.80–2.63, *p* = 0.217) significantly predicted time to closure in the adjusted model. The proportional hazards assumption was met for all covariates via Schoenfeld residual testing. The magnitude of the adjusted hazard ratio for ActiGraft (HR = 10.67) should be interpreted with caution. Effect sizes of this magnitude are uncommon in wound healing intervention studies and may reflect model limitations, including sparse data bias arising from the modest sample size and residual confounding by unmeasured variables, despite the proportional hazards assumption being formally satisfied. Similar considerations apply to the Rigenera HR (4.12), although its magnitude is more consistent with reported treatment effects in regenerative wound care literature.

### 3.4. Sensitivity and Stratified Analyses

Wound Size-Stratified Analysis (Table 3): For wounds measuring <10 cm^2^ (where the groups were most comparable in baseline characteristics), ActiGraft achieved a 71.4% closure rate (15/21) versus advanced dressings at 37.5% (6/16; *p* = 0.043) and Rigenera at 52.9% (9/17). For wounds measuring 10–20 cm^2^, the closure rates were similar across groups (advanced: 52.9%; ActiGraft: 66.7%; Rigenera: 66.7%). No ActiGraft patients had wounds > 20 cm^2^, precluding comparisons in this stratum. Advanced dressings patients with wounds > 20 cm^2^ achieved 33.3% closure (11/33).

CRF-Stratified Analysis: Among patients without CRF (n = 89), the closure rates were as follows: advanced: 32.1% (17/53); ActiGraft: 66.7% (12/18); and Rigenera: 55.6% (10/18). Among patients with CRF (n = 43), the closure rates were more similar: advanced: 71.4% (10/14); ActiGraft: 71.4% (10/14); and Rigenera: 66.7% (10/15). The paradoxically higher closure rate in the CRF-positive advanced dressings subgroup (71.4%) compared to CRF-negative patients (32.1%) suggests complex confounding structures within this non-randomized cohort, which deserve further investigation.

### 3.5. Wound Area Reduction

Autologous therapies demonstrated faster early wound area reduction. At 12 weeks, the mean percentage reduction was 4.1% ± 27.9% (advanced), 20.6% ± 17.1% (ActiGraft), and 25.9% ± 23.1% (Rigenera). At 52 weeks, they were as follows: advanced: 81.8% ± 25.8%; ActiGraft: 95.0% ± 8.5%; Rigenera: 88.5% ± 18.3%. The low variance in the ActiGraft group at 52 weeks reflects the high proportion of completely closed wounds. Percentage reduction was not adjusted for the baseline wound area in this descriptive analysis; given the significant baseline differences, direct comparisons of percentage reduction across groups should be interpreted with caution.

### 3.6. Complications

Wound infections requiring systemic antibiotics occurred in eight patients (11.9%) in advanced dressings, 0 (0%) in ActiGraft, and one (3.0%) in Rigenera. The zero infection rate in the ActiGraft group (0/32) corresponds to a one-sided 97.5% upper confidence bound of 9.4% (rule of three: 3/n), indicating that while no infections were observed, the true infection rate could be as high as 9.4%. The combined autologous therapies versus the advanced dressings group exhibited Fisher’s exact *p* = 0.006. All infections in the advanced dressings group were local cellulitis and managed with oral antibiotics; none of the patients required hospitalization or intravenous therapy. The single Rigenera infection was a superficial wound infection treated with oral antibiotics. The zero infection rate in the ActiGraft group—while clinically notable—should be interpreted in the context of smaller wound sizes in this group, as larger wounds carry inherently higher infection risk. No major amputations (above-ankle) occurred in any group during the 52-week follow-up. No procedure-related adverse events were reported in the autologous therapy groups.

## 4. Discussion

This retrospective real-world cohort study found that autologous blood clot therapy (ActiGraft) and autologous micrograft therapy (Rigenera) were associated with higher unadjusted wound closure rates and significantly faster healing compared to advanced dressings in patients with refractory chronic lower limb ulcers. However, important caveats temper these findings. After multivariable adjustment for baseline wound area, chronic renal failure, age, and peripheral vascular disease, the closure rate advantage was substantially attenuated and did not reach statistical significance, consistent with confounding by indication in this non-randomized design. The time-to-healing advantage, assessed using Cox regression, remained highly significant after adjustment (ActiGraft HR = 10.67, Rigenera HR = 4.12), suggesting a genuine acceleration of healing trajectories independent of baseline differences.

The substantial baseline wound size imbalance (advanced dressings median of 20.0 cm^2^ vs. autologous therapies median of 9.0 cm^2^) represents the most important limitation of this study and likely explains much of the unadjusted treatment effect. Multiple sensitivity analyses addressed this concern. Wound size-restricted analyses limited to wounds measuring <10 cm^2^ (where groups were most comparable) showed a consistent direction of effect: a closure rate 71.4% for ActiGraft vs. 37.5% for advanced dressings. This within-stratum comparison—while limited by sample size—provides more credible evidence than the overall comparison. No ActiGraft patients had wounds measuring >20 cm^2^ (compared to 49.3% of advanced dressings patients), indicating that systematic selection by wound size confounds the primary analysis. The patient-choice design likely reflects both clinical judgment (offering advanced therapies to patients perceived as good candidates) and patient motivation, creating confounding by indication that cannot be fully addressed using statistical adjustments [29].

The chronic renal failure distribution presents a complex confounding pattern. CRF was more prevalent in autologous therapy groups (44–46%) versus controls (21%), representing a negative prognostic factor that would be expected to bias against autologous therapies. However, CRF-stratified analysis revealed paradoxically higher closure rates in CRF-positive controls (71.4%) compared to CRF-negative controls (32.1%), while autologous therapy closure rates were consistent regardless of the CRF status (~67–71%). This suggests that the CRF-positive control patients may represent a distinct subgroup with favorable wound characteristics beyond what is captured using measured covariates, highlighting the limitations of observational data in establishing causality.

The mechanistic distinction between the two autologous therapies may partly explain the differential outcomes. ActiGraft functions as a biologic dressing delivering platelet- and leukocyte-derived growth factors within a fibrin scaffold, and it is mechanistically comparable to PRP products but without concentration enrichment [19,20]. Its repeated application protocol (4–8 weekly sessions) provides sustained growth factor delivery, maintaining a prolonged anabolic wound environment. By contrast, Rigenera delivers viable progenitor cells in a single application, providing direct cellular replenishment but without sustained dosing [21,22]. The superior unadjusted outcomes for ActiGraft over Rigenera may reflect the benefit of repeated application rather than an inherent mechanistic advantage; alternatively, it may reflect unmeasured differences in patient selection between these groups. Without a biological characterization of the applied products (growth factor concentrations for ActiGraft, viable cell counts, and progenitor cell quantification for Rigenera), it is not possible to correlate treatment dose with clinical response, which represents an important limitation.

Compared to published evidence, the observed ActiGraft closure rate of 68.8% aligns with a recent randomized trial by Snyder et al. (2024) reporting 51% closure for ActiGraft versus 18% for standard care in DFUs at 16 weeks [23]. The Rigenera closure rate of 60.6% is consistent with pilot data from Baglioni et al. (2024) demonstrating significant wound reduction with autologous micrografts [24]. The advanced dressing closure rate of 40.3% aligns with the expected outcomes for refractory wounds in clinical practice [5,6]. The NNT of 3.5 (unadjusted) for ActiGraft compares favorably to other wound healing interventions, although this figure should be interpreted with caution given the baseline imbalances.

The lower infection rate in the autologous therapy groups (0–3.0% vs. 11.9%; Fisher’s exact *p* = 0.006) is clinically notable but must be interpreted considering the confounded baseline differences. Larger wounds carry inherently greater infection risk; thus, the infection rate difference may reflect wound size rather than a direct protective effect of autologous therapies. Adjusted analysis was limited by the zero infection rate in the ActiGraft group, precluding logistic regression for this comparison.

From a healthcare resource perspective, this study did not perform a formal cost-effectiveness analysis, which is acknowledged as a limitation. Preliminary estimates from the published literature suggest ActiGraft per-application costs of approximately $300–500, with total treatment costs of $1500–4000 for a complete 4–8 session course. Rigenera costs approximately $2000–3000 for the single-application procedure. Advanced dressings typically cost $100–300 in total materials over the treatment course, although indirect costs of prolonged non-healing (outpatient visits, nursing time, hospitalizations, and potential amputation) are substantially higher [30]. Formal health economic analysis, including incremental cost-effectiveness ratios, cost per additional healed wound, and quality-adjusted life year analysis, should be performed in future studies to inform value-based clinical decision-making.

This study has several important limitations. First, the non-randomized, patient-choice design introduces confounding by indication that cannot be fully addressed by statistical adjustment. Second, the significant baseline wound size imbalance represents the most critical confounder and likely inflates unadjusted treatment effects. Third, outcome assessors were not blinded to treatment allocation, introducing potential detection bias; however, all assessments used standardized digital photography with independent dual review. Fourth, this study was not pre-registered, and the results should be considered hypothesis-generating. Fifth, no biological characterization of ActiGraft (growth factor concentrations and platelet counts) or Rigenera (viable cell counts and progenitor cell quantification) was performed, precluding mechanistic interpretation. Sixth, a cost-effectiveness analysis was not performed. Seventh, the single-center design limits generalizability beyond the Israeli healthcare system. Eighth, the narrow SINBAD score range (5–6) limits generalizability to less severe wounds. Ninth, wound etiology was not stratified (neuropathic vs. venous vs. mixed), and different etiologies may respond differently to autologous therapies.

Future studies should prioritize adequately powered randomized controlled trials comparing ActiGraft, Rigenera, and standard care with stratification by wound size, etiology, and severity. Such trials should include the biological characterization of treatment products, formal cost-effectiveness analyses, quality-of-life assessments, and longer-term recurrence outcomes. Pre-registration with a statistical analysis plan would strengthen causal inference.

## 5. Conclusions

In this retrospective cohort study of 132 patients with refractory chronic lower limb ulcers, autologous blood clot therapy (ActiGraft) was associated with a higher unadjusted wound closure rate (68.8% vs. 40.3%; RR = 1.71, *p* = 0.015) and significantly faster healing (adjusted HR = 10.67, *p* < 0.001) compared to advanced dressings. Autologous micrograft therapy (Rigenera) showed intermediate unadjusted results (60.6%; *p* = 0.089) with significantly accelerated healing (adjusted HR = 4.12, *p* = 0.001). After multivariable adjustments for baseline wound area and other covariates, the closure rate differences were attenuated and no longer significant (ActiGraft adjusted RR = 1.38, *p* = 0.179), consistent with confounding by indication from substantial wound size imbalances. Both autologous therapies were associated with lower infection rates (0–3.0% vs. 11.9%). These hypothesis-generating findings from a non-randomized cohort with significant baseline imbalances warrant confirmation in adequately powered randomized controlled trials with stratification by wound characteristics and formal biological and economic evaluation.

## Figures and Tables

**Table 1:** Baseline characteristics by treatment group.

Variable	Advanced Dressings (n = 67)	ActiGraft (n = 32)	Rigenera (n = 33)	*p*-Value
**Age, years (mean ± SD)**	62.6 ± 11.4	64.9 ± 12.2	66.8 ± 11.7	0.237
**Male sex, n (%)**	38 (56.7)	20 (62.5)	19 (57.6)	0.254
**Diabetes, n (%)**	64 (95.5)	32 (100)	32 (97.0)	0.362
**PVD, n (%)**	26 (38.8)	13 (40.6)	16 (48.5)	0.622
**CRF, n (%)**	14 (20.9)	14 (43.8)	15 (45.5)	0.014
**Wound area, cm^2^ (median, IQR)**	20.0 (8.0–33.0)	9.0 (6.5–9.5)	9.0 (6.0–12.0)	<0.001
<10 cm^2^, n (%)	16 (23.9)	21 (65.6)	17 (51.5)	
10–20 cm^2^, n (%)	18 (26.9)	11 (34.4)	12 (36.4)	
>20 cm^2^, n (%)	33 (49.3)	0 (0)	4 (12.1)	
**Wound depth, mm (mean ± SD)**	3.1 ± 0.9	2.8 ± 0.8	2.9 ± 0.7	0.245
**SINBAD score (mean ± SD)**	5.1 ± 0.4	5.0 ± 0.0	5.0 ± 0.2	0.184
SINBAD = 5, n (%)	57 (85.1)	32 (100)	32 (97.0)	
SINBAD = 6, n (%)	10 (14.9)	0 (0)	1 (3.0)	

PVD: Peripheral vascular disease; CRF: chronic renal failure; IQR: interquartile range; SINBAD: Site, Ischemia, Neuropathy, Bacterial infection, Area, Depth. Bold *p*-values indicate *p* < 0.05.

**Table 2:** Wound closure outcomes at 52 weeks.

Group	Closure n (%)	Unadj. RR (95% CI)	*p*-Value	Adj. RR (95% CI)	Adj. *p*	ARR (%)	NNT
Adv. Dressings	27/67 (40.3%)	Ref	—	Ref	—	—	—
ActiGraft	22/32 (68.8%)	1.71 (1.17–2.48)	0.015	1.38 (0.86–2.21)	0.179	28.5	3.5
Rigenera	20/33 (60.6%)	1.50 (1.01–2.25)	0.089	1.19 (0.73–1.94)	0.488	20.3	4.9
Combined	42/65 (64.6%)	1.60 (1.13–2.27)	0.009	1.28 (0.83–1.98)	0.270	24.3	4.1

RR: Relative risk; NNT: number needed to treat; CI: confidence interval; Adj.: adjusted for log (wound area), CRF, age, and PVD. Bonferroni-corrected threshold: *p* < 0.0167. Combined analysis is pre-specified exploratory.

**Table 3:** Stratified analysis by baseline wound size.

Wound Size	Adv. Dressings	ActiGraft	Rigenera
<10 cm^2^	6/16 (37.5%)	15/21 (71.4%)	9/17 (52.9%)
10–20 cm^2^	9/17 (52.9%)	4/6 (66.7%)	6/9 (66.7%)
>20 cm^2^	11/33 (33.3%)	N/A (0 patients)	3/4 (75.0%)

Values are n/N (%). N/A: No patients in this stratum.

## Data Availability

The de-identified dataset is available in Supplementary Materials. Additional data are available from the corresponding author upon reasonable request.

## Supplementary Materials

The following supporting information can be downloaded at https://www.mdpi.com/article/10.3390/jcm15051902/s1.
